# Gut Microbiota and Its Metabolites: The Emerging Bridge Between Coronary Artery Disease and Anxiety and Depression?

**DOI:** 10.14336/AD.2024.0538

**Published:** 2025-06-19

**Authors:** Haiyang Chen, Lijun Zhang, Yanwei Li, Xiangxi Meng, Yunpeng Chi, Meiyan Liu

**Affiliations:** ^1^Department of Psycho-cardiology, Beijing Anzhen Hospital, Capital Medical University, Beijing, China.; ^2^School of Clinical Medicine, Henan University, Kaifeng, China.; ^3^School of Traditional Chinese Medicine, Beijing University of Chinese Medicine, Beijing, China.

**Keywords:** coronary artery disease, anxiety and depression, gut microbiota, gut microbiota-derived metabolites

## Abstract

The increasing studies indicated that cardiovascular diseases, such as coronary artery disease (CAD), usually induce and exacerbate psychological problems, including anxiety and depression. These psychological issues are admitted as independent risk factors of heart disease as well. The interaction between CAD and anxiety and depression deteriorates the development and prognosis of CAD, which severely threatens the quality of life of patients. Although the existing mechanisms revealed the pathological relationship between CAD and anxiety and depression, there are few studies investigating the correlation between CAD and anxiety and depression from the aspect of gut microbiota (GM) and its metabolites. Therefore, in this review, we summarized whether GM and its metabolites are the emergent bridge between CAD and anxiety and depression. The results showed that there are four kinds of jointly up-regulated bacteria (i.e., *Staphylococcus*, *Escherichia coli*, *Helicobacter pylori*, and *Shigella*) and five kinds of jointly down-regulated bacteria (i.e., *Prevotella*, *Lactobacillus*, *Faecalibacterium prausnitzii*, *Collinsella*, and *Bifidobacterium*) in CAD as well as anxiety and depression. In addition, in CAD and anxiety and depression, the dysbiosis of the former four kinds of bacterium frequently leads to the outburst of inflammatory response, and the dysbiosis of the latter five kinds of bacterium is usually related to the metabolic abnormality of short-chain fatty acids, bile acids, and branched-chain amino acids. Therefore, we believe that GM and its metabolites act as the emergent bridge between CAD and anxiety and depression. The findings of this review provide novel insights and approaches for the clinical treatment of patients with both CAD and anxiety and depression.

## 1. Introduction

With the expedited tempo of life and the worsening trend of aging, the prevalence of coronary artery disease (CAD) and some psychiatric disorders, such as anxiety and depression, are becoming increasingly severe. CAD, one chronic lifelong disease accompanied by multiple complications, gradually forms the dominant psychological stressor for patients to induce psychiatric disorders such as anxiety and depression [1, 2]. Meanwhile, psychiatric disorders, including anxiety and depression, make adverse contributions to the progression and prognosis of CAD and profoundly threaten the lives of patients [3]. However, there are still some limitations regarding the precise clinical diagnosis and treatment for CAD with anxiety or depression until now. Although the clinically applied approaches, such as echocardiography, electrocardiogram, and biomarkers (including creatine kinase MB and cardiac troponin I), reliably diagnose CAD, they can’t estimate patients’ psychiatric disorders [4, 5]. Moreover, one of our previous meta-analysis studies demonstrated that clinically adopted cardiovascular drugs, such as β-blockers, calcium channel blockers, diuretics, and nitrate esters, may induce psychiatric disorders, including anxiety and depression, in patients in different degrees [6]. Therefore, there is an urgent need to investigate the relative pathological mechanisms of CAD with anxiety or depression in greater detail to overcome the existing bottlenecks. Fortunately, more and more studies manifested that the regulation of gut microbiota (GM) is not only beneficial to mitigating cardiovascular diseases and psychiatric disorders but also may be proposed as the potential biomarker for these diseases, providing novel possibilities for the clinical diagnosis and treatment of patients of CAD with anxiety or depression [7, 8].

The human gut, an actively “metabolic superorganism”, is one of the most existing complicated biological systems [9]. The total number of GM parasitized in humans exceeds 10^14^, which is more than ten times the total number of human cells [10]. The GM dynamically integrates signals from the host and environment to maintain physiological homeostasis by facilitating digestion and absorption, inhibiting the growth of pathogens, and enhancing immune responses [11]. Increasingly studies demonstrated that the derangement of the relative abundance and diversity of the GM and the alteration of gut metabolites frequently are associated with the generation and progression of multiple disorders, such as diverse cancers, cardiovascular and cerebrovascular diseases, anxiety, depression, and Alzheimer’s disease, etc. [12-16]. The previous studies proved that the level of GM in CAD was significantly altered, among which the number of *Firmicutes* and *Clostridium* increased dramatically and the number of *Bacteroidetes* and *Proteobacteria* decreased prominently [17-19]. Moreover, the previous study also confirmed that trimethylamine N-oxide, one of the intestinal metabolites, promotes atherosclerosis by suppressing cholesterol metabolism to induce platelet aggregation and thrombosis, indicating that intestinal metabolites are involved in the progression of CAD [20]. Meanwhile, some previous studies also suggested that the level of *Bacteroidetes*, *Proteobacteria*, and *Actinobacteria* increased but the level of *Firmicutes* decreased in patients with anxiety and depression when compared with the healthy control group [15, 21]. It follows that the regulation of GM is involved in the progression of CAD, anxiety, and depression.

Although multitudinous previous studies investigated the effects of GM respectively on CAD, anxiety, and depression, the studies exploring the association between CAD and anxiety, or depression based on the alteration of GM are extremely few. Therefore, this review summarizes the role of GM and its metabolites in the progression of CAD, anxiety, and depression, analyzes and discusses the possibility of considering GM as a potential therapeutic target for CAD combined with anxiety or depression, providing a novel perspective for the clinical treatment of CAD combined with anxiety or depression.

## 2. Effects of GM on CAD

The *Firmicutes* and *Bacteroidetes* are the main proportions of microbiota, whose ratio is considered one of the evaluation indicators for the emergence of diseases, especially for cardiovascular disorders [22]. Although the proportion and composition of GM consistently change during the progression of CAD, the alteration of GM in CAD patients is dominantly reflected in the elevation of *Firmicutes* and the decline of *Bacteroidetes* [23]. Moreover, previous studies also indicated that the abnormal change in *Proteobacteria* and *Actinobacteria* was also associated with CAD [24, 25].

After reviewing related literature, we found that although different microbiota at the genus level of *Bacteroidetes* that were involved in CAD all declined, mainly including *Bacteroides vulgatus*, *Bacteroides dorei*, *Bacteroides fragilis*, *Bacteroides ovatus*, *Prevotella*, and *Alistipes*, the concerned mechanisms of them were different. One of the extensively accepted viewpoints was that the decreased level of *Bacteroides vulgatus* and *Bacteroides dorei* aggravated the CAD by promoting the production of endotoxin and lipopolysaccharide (LPS) to facilitate inflammatory responses [26]. Moreover, some previous studies demonstrated that the elevation of the level of *Streptococcus*, *Staphylococcus*, and *Ruminococcus*, three kinds of *Firmicutes*, was highly related to the formation of coronary atheromatous plaque and hyperlipidemia, which are the dominant risk factors of CAD [27-29]. Although the increased *Firmicutes* was positive for the progression of CAD, the elevation of some other microbiota at the genus level of *Firmicutes*, such as *Lactobacillus*, may contribute to preventing CAD [18]. One prospective and randomized controlled study demonstrated that *Lactobacillus* reduced the serum total cholesterol and low-density lipoprotein levels in elderly patients, which were the risk factors for CAD [30]. Moreover, some studies indicated that the lower level of *Faecalibacterium prausnitzii* may implicate the high risk and poor prognosis of CAD [31, 32]. One previous study reported that *Faecalibacterium prausnitzii* mitigates cardiovascular risk factors by promoting the production of butyrate [33]. Furthermore, it has affirmed that *Ochrobactrum anthropi*, *Ralstonia pickettii*, *Escherichia coli*, *Helicobacter pylori*, and *Escherichia-Shigella*, which are all belonging to *Proteobacteria*, were all significantly elevated in CAD [34-37]. The elevation of *Escherichia coli*, *Helicobacter pylori*, and *Shigella* damages the intestinal mucosal barrier to facilitate LPS entering the bloodstream to activate inflammatory signaling pathways and enhance the release of pro-inflammatory cytokines, thereby exacerbating CAD [38, 39]. Meanwhile, it was attested that *Collinsella* and *Bifidobacterium*, two kinds of *Actinobacteria*, were dramatically decreased in CAD [40, 41]. The disorders of *Collinsella* and *Bifidobacterium* lead to the metabolic abnormality of bile acid to induce dyslipidemia, increasing the risk of CAD [6, 42]. The tendencies and relative mechanisms of different microbiota at the genus level involved in CAD are listed in Table 1.

**Table 1:** The changing trend and relative mechanisms of different bacteria at the genus level that were involved in CAD.

Phylum level	Genus level	Changing trend of bacteria in CAD	Mechanism of bacteria on CAD	Ref.
***Bacteroides***	*Bacteroides vulgatus*	Decrease	Promoting the production of endotoxin and lipopolysaccharide to aggravate inflammatory responses.	[26]
	*Bacteroides dorei*	Decrease	Promoting the production of endotoxin and lipopolysaccharide to aggravate inflammatory responses.	[26]
	*Bacteroides fragilis*	Decrease	Declining the expression level of Forkhead box protein P3 in Treg cells to promote inflammatory responses.	[212]
	*Bacteroides ovatus*	Decrease	Elevating the serum level of multiple cardiac-toxic metabolites.	[213]
	*Prevotella* ^*^	Decrease	Decreasing the production of SCFAs to increase the level of cholesterol.	[214]
	*Alistipes*	Decrease	Influencing the level of serum/urinary/fecal metabolites.	[215]
***Firmicutes***	*Streptococcus*	Increase	Promoting the mRNA expression level of proinflammatory cytokines in the aorta.	[216]
	*Staphylococcus* ^*^	Increase	Inducing endocarditis to aggravate coronary thrombosis.	[217]
	*Lactobacillus* ^*^	Decrease	Causing leptin resistance to exacerbate obesity, one risk factor of CAD.	[218]
	*Faecalibacterium prausnitzii* ^*^	Decrease	Aggravating inflammation to assist atherosclerosis.	[31]
	*Ruminococcus*	Increase	Promoting the release of inflammatory cytokines.	[219]
***Proteobacteria***	*Ochrobactrum anthropi*	Increase	Inducing septicaemia to exacerbate left ventricular dysfunction.	[220]
	*Ralstonia pickettii*	Increase	Causing the decline of the level of unsaturated fatty acid to accelerate the progression of CAD.	[34]
	*Escherichia coli* ^*^	Increase	Increasing inflammatory response and the level of adiponectin as well as disturbing glycolipid metabolism.	[25]
	*Helicobacter pylori* ^*^	Increase	Activating the expression of pro-inflammatory factors.	[221]
	*Shigella* ^*^	Increase	Inhibiting the biosynthesis of bile acids to induce the accumulation of cholesterol.	[222]
***Actinobacteria***	*Collinsella* ^*^	Decrease	Inducing metabolic disorders to affect the biosynthesis of ALT and AST.	[223]
	*Bifidobacterium* ^*^	Decrease	Increasing the biosynthesis of methylxanthine, malonate, and trimethylamine-N-oxide.	[71]

* Bacteria with the same changing trend as anxiety and depression.

## 3. Effects of GM-derived metabolites on CAD

### 3.1 Effects of GM-derived trimethylamine/ trimethylamine N-oxide on CAD

More and more studies suggested that trimethylamine N-oxide (TMAO) and its metabolite precursor, trimethylamine (TMA), were independent risk factors for CAD [20]. Choline converted from phosphatidylcholine in diet combines with L-carnitine to further transform into TMA through microbial metabolism in the intestine [43]. One study on the metagenomic association analysis suggested that the abundance of GM involved in TMA synthesis was significantly increased in patients with CAD [44]. The microbiome-derived TMA further metabolizes into TMAO through flavin-dependent monooxygenase in the liver, kidneys, and other tissues [45]. TMAO promotes atherosclerosis and the rupture of unstable plaque by not only triggering oxidative stress but also promoting the liberation of inflammatory cytokines to exacerbate the inflammatory response [46-48]. Moreover, TMAO facilitated the formation of a thrombus by not only declining the expression of thrombomodulin in endothelial cells to enhance procoagulant activity but also triggering the release of calcium ions in platelets to induce platelet hyperreactivity [49-51]. It was found that the ratio of *Firmicutes* to *Bacteroidetes* increased in individuals with higher serum TMAO levels, while the ratio of *Firmicutes* to *Bacteroidetes* decreased in individuals with serum lower TMAO levels [52]. Some previous studies also suggested that the elevation of beneficial microbiota, such as *Lactobacillus*, effectively declines the serum level of TMAO [53, 54]. Furthermore, out of the independent predictive ability of TMAO for AMI, the GM related to TMAO level may serve as the potential biomarkers for the diagnosis of CAD [55].

### 3.2 Effects of GM-derived short-chain fatty acids on CAD

Multiple short-chain fatty acids (SCFAs) metabolized by GM contribute to maintaining intestinal immune homeostasis to regulate lipid and glucose metabolism, which includes acetic acid, propionic acid, and butyric acid [56]. Different SCFAs are derived from different microbiota, in which acetic acid and propionic acid are dominantly metabolized from *Bacteroidetes* and butyric acid is predominantly metabolized from *Firmicutes* [57]. Indole-3-propionic acid, a metabolite of *Clostridium sporogenes*, has been affirmed to suppress atherosclerosis by promoting reverse cholesterol transport [58, 59]. Acetic acid, the most abundant SCFA in the peripheral circulation, improves glucose homeostasis and regulates inflammatory response by inhibiting adipocyte lipolysis and promoting fat oxidation [60]. The disorder of the relative abundance of some microbiota in *Firmicutes* in CAD frequently causes the metabolic abnormality of SCFAs. Butyrate, one metabolite that depends on *Faecalibacterium prausnitzii*, suppresses macrophage activation and reduces the production of pro-inflammatory cytokines to alleviate CAD by enhancing the intestinal barrier to prevent LPS translocation [61, 62]. Moreover, the reduction of the relative abundance of *Lactobacillus*, one kind of beneficial bacteria, also leads to a decrease in the generation of SCFA, thereby affecting energy metabolism and the prognosis of CAD patients [63].

### 3.3 Effects of GM-derived bile acids on CAD

Cholesterol, one of the risk factors for atherosclerotic plaque formation, is the dominant raw material for the biosynthesis of primary bile acids (BAs) that promote the absorption of lipid substances [64]. The formed primary BAs are uncoupled by gut bacteria and bile salt hydrolytic enzymes to produce secondary BAs [65]. One previous study found that the level of primary bile acids decreased and the level of secondary bile acids, especially lithocholic acid, increased in patients with cardiovascular diseases [66]. The dysbiosis of GM with the bile-salt hydrolase activity, including *Bacteroides*, *Lactobacillus*, and *Bifidobacterium*, leads to the accumulation of free BAs, such as chenodeoxycholic acid and deoxycholic acid, thereby inducing the disorder of lipid and glucose metabolism to enlarge atherosclerotic plaque and increase the risk of CAD [67, 68]. In addition, GM-derived BAs, such as lithocholic acid and deoxycholic acid, activate farnesol X receptor to suppress the expression of cholesterol 7 α hydroxylase, which results in the increase in the level of cholesterol and finally leads to the formation of atherosclerotic plaque [69]. Moreover, the transplantation of *Clostridium symbiosum* and *Eggerthella* genus from the feces of CAD mice to normal ones intervened in the normal metabolism of BAs to lead to an elevation of circulating cholesterol [70].

### 3.4 Effects of GM-derived branched-chain amino acids on CAD

Branched-chain amino acids (BCAAs) are essential to regulate the balance of GM and facilitate the metabolism of the GM for the intracorporal amino acids, whose abnormal metabolism is associated with the progression of CAD as well [71, 72]. It was reported that BCAAs induced mitochondrial dysfunction by activating the mammalian target of the rapamycin signaling pathway, resulting in cardiomyopathy [73]. Moreover, it was demonstrated that the dysbiosis of some microbiota in *Bacteroidetes*-induced increased BCAAs was related to insulin resistance, which is also associated with CAD [74]. The results of the combination analysis between serum metabolomics and intestinal microbiome indicated that *Prevotella* and *Bacteroides* caused insulin resistance by elevating the level of BCAAs in serum [75]. The intermediate metabolite of valine, 3-hydroxyisobutyric acid, participates in regulating the transport of fatty acids to promote the accumulation of fat in the muscle, resulting in insulin resistance [76]. Besides, some aromatic amino acids, such as phenylalanine, histidine, and tryptophan, affect the progression of CAD. With the help of GM, one of the metabolites of phenylalanine, p-cresyl sulfate, not only injures endothelial cells and smooth muscle cells through oxidative stress pathways but also serves as a uremic toxin to increase the risk of CAD [77]. Imidazole propionate, one of the products of histidine metabolized by GM, was proven to suppress glucose metabolism through the MAPK signaling pathway to aggravate CAD [78]. Although the majority of BCAAs and aromatic amino acids are adverse to CAD, indole and its derivatives, such as 3-indole-3-propionic acid, 3-indole-3-ethanol, indole-3-acrylic acid, that are converted by tryptophan with the help of *Bacteroides*, *Bifidobacterium*, and *Streptococcus* possess anti-inflammatory activity and protect against CAD [79].

### 3.5 Effects of GM-derived lipopoly-saccharide on CAD

LPS, the dominant component in the cell wall of Gram-negative bacteria, is released into the bloodstream to cause systemic inflammation and sepsis after the death and lysis of GM [80]. LPS not only promotes major adverse cardiac events by increasing platelet activation but also elevates blood pressure by inducing an inflammatory response [81, 82]. In addition, the released LPS combines with the CD14 receptor to activate the NF-κB signaling pathway to promote an inflammatory response to form atherosclerosis [83].

## 4. Effects of GM on anxiety and depression

Anxiety and depression are usually accompanied by the dysbiosis of GM, indicating that GM may be the main interventional factor for the progression of anxiety and depression [84]. The results of the behavior experiment indicated there is less anxiety-like behavior exhibited in the sterile mice, but the anxiety-like behavior is gradually frequent in sterile mice after cohabiting with the mice with normal GM [85]. Moreover, the comparative analysis of the GM between anxiety patients and normal ones suggested that the *Firmicutes* was significantly decreased and the proportion of *Bacteroidetes* was dramatically increased in the anxiety patients [86]. Furthermore, one previous study demonstrated that the elevation of the level of conditionally pathogenic bacteria, such as *Proteobacteria* and *Actinobacteria* was one of the significant factors to induce anxiety and depression-like behaviors as well [87]. It can be seen that the imbalance of GM is closely correlated to the progression of anxiety and depression.

Although it was discovered that the *Alistipes* and *Bacteroidetes* genus, two different kinds of bacteria in the *Bacteroidetes* phylum both elevated in anxiety and depression patients, the level of another kind of bacteria belonging to *Bacteroidetes* phylum, *Prevotella*, declined in the anxiety and depression patients [88-90]. Multiple previous studies demonstrated that the *Alistipes*, an indole-positive organism with the availability to decrease serotonin, significantly elevated in both mice and humans with anxiety and depression [91, 92]. Although the *Bacteroides* genus was usually considered beneficial bacteria to suppress inflammation and produce SCFAs, such as acetate and propionate, it was always discovered that the level of the *Bacteroides* genus elevated in subjects with anxiety and depression [90, 93]. It was explained that the elevation of the *Bacteroides* genus in anxiety and depression may originate from a compensatory mechanism to prevent further deterioration of neurological damages [90]. It was reported that a lower abundance of bacteria with anti-inflammatory effects, such as *Prevotella*, was found in common brain diseases, including anxiety and depression [94]. Recent clinical and animal studies indicated that the relative abundance of *Lactobacillus*, one kind of bacteria belonging to *Firmicutes*, usually declined in subjects with anxiety and depression [95, 96]. The supplement of *Lactobacillus* kept the nervous system homeostasis by alleviating inflammatory response, for example, *Lactobacillus* promoted the formation of indole-3-aldehyde to activate the AHR gene and stimulated the secretion of anti-inflammatory factor, IL-2 [97]. Interestingly, the previous study discovered that the level of *Streptococcus* in hosts with anxiety was up-regulated but down-regulated in hosts with depression [98]. Moreover, mice with anxiety and depression-like behaviors were characterized by higher abundances of *Staphylococcus* compared with the normal ones [99]. *Faecalibacterium prausnitzii* is one of the other important bacteria that belong to *Firmicutes*, whose level was demonstrated to decline in patients with anxiety and depression through clinically comparative analysis [100]. In addition, the relative abundance of one potential beneficial bacterium, *Lachnospira*, was reported to be negatively related to anxiety and depression [101, 102]. The level of *Ruminococcus*, one of the most effective bacterial genera for decomposing carbohydrates, was proved to be related to anxiety and depression as well [103, 104]. Furthermore, the previous studies indicated that the levels of *Escherichia coli*, *Helicobacter pylori*, and *Shigella*, the three kinds of bacteria belonging to *Proteobacteria*, all elevate in subjects with anxiety and depression [105-107]. Meanwhile, the relative abundance of *Collinsella* and *Bifidobacterium*, two kinds of bacteria that are subordinate to *Actinobacteria*, both declines in patients with anxiety and depression [108, 109]. The tendencies and relative mechanisms of different microbiota at the genus level involved in anxiety and depression are listed in Table 2.

**Table 2:** The changing trend and relative mechanisms of different bacteria at the genus level that were involved in anxiety and depression.

Phylum level	Genus level	Changing trend of bacteria in anxiety and depression	Mechanism of bacteria on anxiety and depression	Ref.
***Bacteroides***	*Prevotella* ^#^	Decrease	Affecting the amino acid metabolism to cause abnormal dopamine signaling.	[224]
	*Alistipes*	Increase	Decreasing the synthesis of tryptophan to reduce the availability of serotonin.	[21]
	*Bacteroides*	Increase	A compensatory mechanism to prevent further deterioration of neurological damages by inhibiting inflammation and producing SCFAs.	[90, 93]
***Firmicutes***	*Lactobacillus* ^#^	Decrease	Regulating multiple immune-related pathways to disrupt the immune barrier.	[225]
	*Streptococcus*	Controversial	Reducing the levels of pro-inflammatory cytokines/Reducing the synthesis of SCFAs, especially for acetate.	[226]/ [227]
	*Staphylococcus* ^#^	Increase	Inducing neuroinflammation by overexpressing TLR2 accompanied by increasing GLS1 and p-STAT3 expression.	[167]
	*Faecalibacterium prausnitzii* ^#^	Decrease	Declining the level of SCFAs and elevating the level of inflammatory cytokines.	[228]
	*Lachnospira*	Decrease	Suppressing the production of butyrate.	[227]
	*Ruminococcus*	Decrease	Up-regulating oxidative phosphorylation-related genes in mitochondrion and down-regulating neuronal plasticity-related genes.	[229]
***Proteobacteria***	*Escherichia coli* ^#^	Increase	Up-regulating the expression of IL-1β and IL-6 to induce neuroinflammation.	[230]
	*Helicobacter pylori* ^#^	Increase	Activating the mTOR pathway to suppress the secretion of ghrelin to induce pyroptosis and neuroinflammation.	[204]
	*Shigella* ^#^	Increase	Secreting lipopolysaccharides to increase blood-brain barrier permeability to activate neuroinflammation.	[231]
***Actinobacteria***	*Collinsella* ^#^	Decrease	Down-regulating the level of ursodeoxycholic acid to diminish antioxidant, anti-inflammatory, and anti-apoptotic effects.	[232]
	*Bifidobacterium* ^#^	Decrease	Promoting neuroinflammation, declining the synthesis of serotonin, and activating the hypothalamus-pituitary-adrenal axis.	[233]

# Bacteria with the same changing trend as CAD.

## 5. Effects of GM-derived metabolites on anxiety and depression

### 5.1 Effects of GM-derived short-chain fatty acids on anxiety and depression

SCFA, a neuroactive bacterial metabolite of dietary fiber, possesses the function of regulating brain cognition and behavior [110]. After the free SCFAs in the intestine are transported into the bloodstream through monocarboxylate transporters (MCTs), they further cross the blood-brain barrier with the help of MCTs to regulate neuronal function [111]. Moreover, the decline of SCFAs facilitates the release of pro-inflammatory factors to induce systemic inflammation, including neuroinflammation, thereby resulting in the down-regulation of neurotrophic factors in the hippocampus and cerebral cortex to induce anxiety, depression, and cognitive dysfunction [112]. After the binding of propionate to receptors, the neural activity in the caudate nucleus and nucleus accumbens of the brain is weakened, which is presented as a common brain circuit dysfunction in patients with depression [113]. More than that, SCFAs also mitigate depression not only by reducing the expression level of mineralocorticoid receptors and Adreno-corticotropin-releasing factors to alleviate the HPA axis response but also by regulating the expression level of tryptophan hydroxylase 1 to facilitate the biosynthesis of 5-hydroxytryptophan [114, 115]. The regulation of GM contributes to elevating the level of SCFAs to alleviate anxiety and depression. The long-term feeding of high dietary fiber foods to mice, such as fructo-oligosaccharides or galacto-oligosaccharides, regulated the ratio of *Firmicutes*/*Bacteroidetes* to enhance the level of SCFAs, especially for propionate, thereby improving their anxiety and depression-like behaviors [116, 117]. Moreover, previous studies indicated that there are not only less propionic acid, acetic acid, and butyrate but also less beneficial bacteria that produce butyrate, such as *Faecalibacterium* and *Coprococcus*, in depression patients compared with normal ones, in which the level of *Faecalibacterium* has clinical significance for various mental disorders, including depression [88, 118, 119]. Therefore, GM-derived SCFAs may serve as one of the important mediators influencing the emotions of the host.

### 5.2 Effects of GM-derived bile acids on anxiety and depression

At present, BAs and their receptors and transporters have respectively been detected in the brains and central nervous system cells of humans and animals, suggesting that BAs may play an essential role in the signaling of the central nervous system [120, 121]. The alteration of the level of BAs decreases the expression of farnesoid X receptor to reduce the biosynthesis of brain-derived neurotrophic factor (BDNF), which is one of the significant incentives of depression patients [122]. It was found that taurodeoxycholic acid, one of the secondary bile acids, improved anxiety and depression symptoms in mice by binding to the membrane receptor G protein-coupled bile acid receptor 5 to inhibit the level of neuroinflammatory factors, oxidative nitrification, and endoplasmic reticulum stress [123]. In addition, BAs improved anxiety and depression behaviors by promoting the combination of glucagon-like peptide-1 and its receptor to regulate the metabolism of glucose in the liver as well [124]. The reduction of the bacteria that decline the activity of bile salt hydrolase, including *Bifidobacterium* and *Bacteroidetes* genus, and *Lactobacillus*, leads to the disorder of BAs to induce anxiety and depression behaviors [125, 126].

### 5.3 Effects of GM-derived branched-chain amino acids on anxiety and depression

BCAAs, the potential biomarkers for health, were mainly metabolized by *Bifidobacterium* and *Lactobacillus* and played essential roles in protein synthesis, and secretion and release of diverse hormones, such as insulin and growth hormone, which were associated with anxiety and depression as well [127, 128]. It was found that the significantly reduced level of BCAAs in depression was improved by increasingly taking the high protein diet [129]. Although BCAAs are not precursors for synthesizing neurotransmitters, the alteration in their content affects the effectiveness and availability of other amino acids in the brain to indirectly regulate the level of neurotransmitter precursors and the emotional state [130]. One previous study indicated that feeding mice with high-protein foods enhanced the level of BCAAs to elevate the level of BDNF in the hippocampus to alleviate the social avoidance behavior of mice [131]. Moreover, one previous study demonstrated that the concentration of three kinds of BCAAs, valine, leucine, and isoleucine, was significantly negatively correlated with the Hamilton Depression Rating Scale and Beck Depression Scale scores in patients with severe depression [132].

### 5.4 Effects of GM-derived monoamine neurotransmitters on anxiety and depression

Monoamine neurotransmitters, mainly including dopamine (DA), 5-hydroxytryptamine (5-HT), and noradrenaline (NE), contribute to maintaining homeostasis of the internal environment and improving emotional disorders, whose depletion may be one of the potential factors for the emergence of anxiety and depression [133]. DA, one catecholaminergic neurotransmitter synthesized by central and peripheral dopaminergic neurons, plays an important role in the regulation of anxiety and depression [134]. It was found that chronic stress caused changes in neural adaptability to reduce the synthesis and release of DA to induce depression ultimately [133]. Moreover, the activity of dopamine receptor D1 in the ventral tegmental area is also crucial to regulate anxiety and depression, which is negatively related to the progression of anxiety and depression [135]. Although the enhancement of the level of DA contributed to alleviating depression behaviors, excessive activation of DA in the midbrain leads to anxiety as well [136]. The previous studies indicated that the decreased level of many bacteria at the genus level in the *Clostridium* and *Ruminococcaceae* families prompted the decline of the expression of dopamine receptor D2, which might be one of the mechanisms for the inducement of anxiety [137, 138]. 5-HT, the most widely distributed neurotransmitter derived from the raphe nucleus of the brainstem, is closely related to circadian rhythm, sleep, emotional control, food intake, cognitive activity, and other biological functions [139]. It was reported that the level of serum 5-HT might serve as one of the predicted factors for anxiety and depression and the elevation of the level of serum 5-HT was also conducive to improving patients with anxiety and depression [140]. It was affirmed that the down-regulation of the level of multiple GM, including phylum *Proteobacteria*, class *Clostridia*, order *Bacteroidales*, and genus *Dorea*, significantly upregulated and reduced the expression level of tryptophan hydrogenase 1 (TPH1) and 5-HT transporters, respectively, thereby increasing the level of 5-HT in the organism [141]. In addition, the dysbiosis of GM impaired the synthesis of vitamin B6 and tryptophan, indirectly leading to the decline of the level of 5-HT to induce the occurrence and development of anxiety and depression [142]. Apart from the GM, the metabolites of GM, such as acetate, butyrate, and propionate, promote the synthesis and secretion of 5-HT by directly stimulating TPH1 in intestinal chromaffin cells [114, 143]. NE, the dominant neurotransmitter in the sympathetic nervous system, is closely related to human alertness, memory, attention, and acute stress response [144]. It has been demonstrated that the adrenal medulla and the locus coeruleus in the brain are the primary sites for NE biosynthesis and damage to the locus coeruleus in the brain is the prominent cause of depression patients [133]. Multiple bacteria, including *Bacillus spp.*, *Escherichia spp.*, and *Saccharomyces spp.*, promote the production of NE to mitigate anxiety and depression by protecting the locus coeruleus in the brain [145, 146].

### 5.5 Effects of GM-derived brain-derived neurotrophic factors on anxiety and depression

BDNF, a key neurotrophic factor, improves anxiety and depression by regulating the growth of nerve cells and synaptic plasticity [147]. The previous study revealed that the decline in the level of BDNF caused anxiety-like behavior through the zebrafish model [148]. Meanwhile, it was indicated that some anti-anxiety drugs improve anxiety symptoms in rats by increasing the expression level of BDNF mRNA and protein in the cells of rat brains [149]. It was proved that the early colonization of *Lactobacillus* contributed to alleviating anxiety by increasing the level of BDNF in the hippocampus and amygdala [150]. In addition, butyrate is considered a candidate substance for linking GM and the level of BDNF in the brain. A previous animal study suggested that butyrate promotes the expression level of BDNF mRNA and protein in the prefrontal cortex by inhibiting histone deacetylase [151]. Therefore, the regulation of GM that can produce butyrate, such as the *Faecalibacterium prausnitzii* genus, possesses the potential to prevent the production of anxiety and depression.

**Figure 1.**
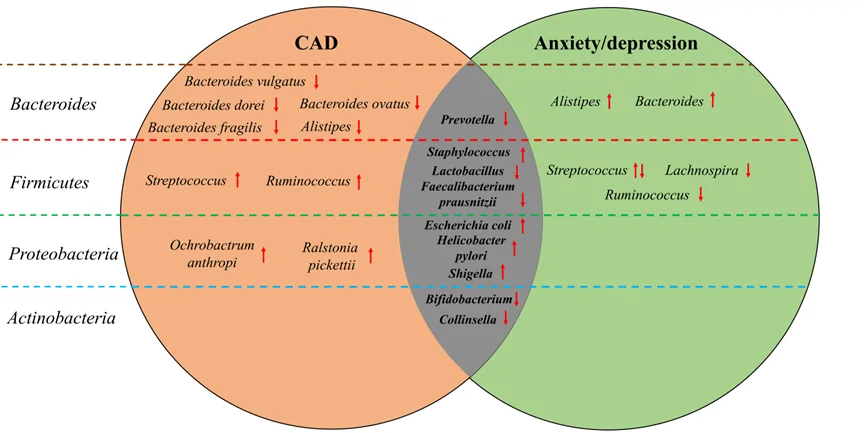
The altered tendency for GM of CAD and anxiety and depression from the four aspects of *Firmicutes*, *Bacteroidetes*, *Proteobacteria*, and *Actinobacteria*.

## 6. Discussion

So far, there are some acknowledged common pathological mechanisms between CAD and anxiety or depression, such as the disorder of the autonomic nervous system and the 5-HT level and the aggregation of the inflammatory response [152, 153]. Anxiety and depression decrease the function of the parasympathetic nervous system to elevate blood pressure and accelerate the heart rate, intensifying cardiac stress [154]. Moreover, the dysfunction of the sympathetic and parasympathetic nerve system further leads to the hyperfunction of the hypothalamus-pituitary-adrenal axis, which causes the ascension of serum cortisol, the abnormality of glucose and lipid metabolism, the enhancement of blood viscosity, the deceleration of blood flow, and the multiplication of the risk of atherosclerosis [155]. In the central nervous system, 5-HT is usually regarded as a neurotransmitter responsible for information transmission between neurons, whose level is crucial to the treatment of anxiety and depression [58, 156, 157]. Furthermore, our previous study also suggested that 5-HT was involved in the treatment of myocardial infarction co-exists with depression by regulating the 5-HT2A receptor on platelets [158]. NLRP3 inflammasome not only is involved in the progression of CAD by aggravating the inflammatory response of myocardial cells but also participates in the occurrence of depression by multiple pathways, especially for the pyroptosis caused by neuroinflammation [147, 159, 160]. Although the above pathological mechanisms strongly attest to the relation between CAD and anxiety or depression, the investigation for the relative mechanisms that are involved in CAD with anxiety or depression at the same time should not stop there. Nowadays, increasing studies indicate that GM plays an essential role in the treatment of CAD, anxiety, and depression [161, 162]. Therefore, this review discussed the relationship between CAD and anxiety, or depression based on a novel perspective, namely GM and its metabolites.

**Figure 2.**
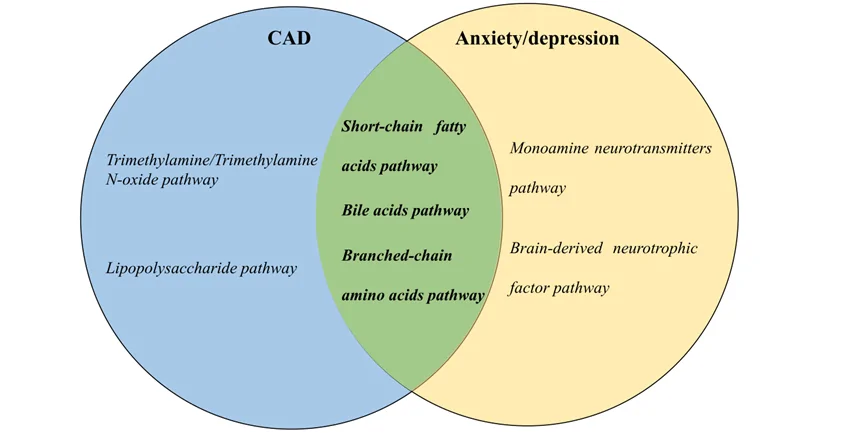
The metabolite pathways of GM of CAD and anxiety and depression.

As Figures 1 and 2 showed, after reviewing the clinical and experimental studies regarding CAD or anxiety and depression based on GM and its metabolites, we marvelously discovered that there are some overlaps between CAD and anxiety and depression on the altered tendency of GM and effects generated by GM-derived metabolites. Generally speaking, the tends of the relative abundance of nine kinds of genus bacteria in both CAD and anxiety and depression are the same, which include *Staphylococcus*, *Escherichia coli*, *Helicobacter pylori*, *Shigella*, *Prevotella*, *Lactobacillus*, *Faecalibacterium prausnitzii*, *Collinsella*, and *Bifidobacterium*. Meanwhile, there are three collective pathways involved in the effects of GM-derived metabolites on CAD or anxiety and depression, including the SCFAs pathway, BAs pathway, and BCAAs pathway. Among the nine kinds of bacteria, the level of the former four kinds, including *Staphylococcus*, *Escherichia coli*, *Helicobacter pylori*, and *Shigella*, is up-regulated but the level of the latter five kinds, embracing *Prevotella*, *Lactobacillus*, *Faecalibacterium prausnitzii*, *Collinsella*, and *Bifidobacterium*, is down-regulated in subjects with CAD or anxiety and depression.

*Staphylococcus* often accumulates into grape clusters, whose representative species is *Staphylococcus aureus*, one zoonotic pathogenic bacterium that induces various infections [163]. *Escherichia coli* and *Helicobacter pylori* are the dominant microbial community of *Proteobacteria*. *Escherichia coli* belongs to conditional pathogenic bacteria, which usually causes gastrointestinal infections or infections of various local tissues and organs [164]. *Helicobacter pylori* usually induce inflammation and immune response to trigger cell degeneration, necrosis, and inflammatory infiltration, whose pathogenicity is dominantly related to the damage to the gastric mucosa by producing toxins [165]. Endotoxins produced by *Shigella* are absorbed by the intestinal tract to further harm the central nervous system and cardiovascular system [166]. The systematic inflammatory response induced by the infection of *Staphylococcus aureus*, *Escherichia coli*, *Helicobacter pylori*, and *Shigella* may be one of the key factors of CAD or anxiety and depression [28, 167-171].

*Prevotella*, the most important microbial community of *Bacteroidetes*, plays a positive role in the degradation of cellulose, proteins, and polysaccharides to decline the level of cholesterol, one risk factor of CAD [172-174]. Moreover, the dominant metabolites of *Prevotella* are SCFAs, such as acetic acid, isobutyric acid, and isovaleric acid [174, 175]. *Lactobacillus*, the most dominant microbial community of *Firmicutes*, plays an essential role in maintaining the health of humans [176]. *Lactobacillus* not only possesses immunomodulatory effects, such as promoting the production of antibodies, activating macrophages, and inducing the production of interferon but also reduces intestinal cholesterol absorption by inhibiting the hydroxymethylglutarate CoA reductase, thereby reducing the cholesterol level [177, 178]. In addition, previous studies suggested that *Lactobacillus* stimulated the production of SCFAs and the excretion of BAs [179, 180]. *Faecalibacterium prausnitzii* is admitted as one of the important producers of SCFAs, especially for butyric acid, and possesses prominent anti-inflammatory activity [181]. The anti-inflammatory effects of *Faecalibacterium prausnitzii* are predominantly reflected in the inhibition of the release of inflammatory cytokines, including interleukins, tumor necrosis factors, and interferons, and the suppression of inflammatory-related pathways, such as NF-κB pathway and NLRP3 pathway, which are the primary pathological mechanism of CAD as well [182, 183]. *Collinsella* is demonstrated to exert anti-inflammatory and antioxidant effects by promoting the synthesis of ursodeoxycholic acid to improve the material and energy metabolism of the host [184]. Moreover, it was also confirmed that the regulation of *Collinsella* on blood lipid metabolism, such as serum total cholesterol, triglycerides, extremely low-density lipoprotein, and high-density lipoprotein cholesterol, was involved in its inflammatory regulatory effects [185]. *Bifidobacterium*, as the foremost microbial community of *Actinobacteria*, is similar to *Lactobacillus*, which both are beneficial microorganisms for the health of the host [186]. It has been confirmed that *Bifidobacterium* prevents the occurrence of cardiovascular adverse events by regulating the metabolism of BAs to decline blood lipid levels to alleviate atherosclerosis [187]. Meanwhile, *Bifidobacterium* gradually exhibits outstanding performance in enhancing neurological function, which exerts anti-depressant and anti-anxiety effects by promoting the generation and release of various neurotransmitters, such as serotonin, dopamine, norepinephrine, and γ-aminobutyric acid [188]. It follows that the dysbiosis of those GM is frequently related to the unbalance of synthesis and metabolism of its metabolites, especially for SCFAs, BAs, and BCAAs.

It was demonstrated that SCFAs alleviate CAD by reducing lipid accumulation, declining blood pressure, inhibiting inflammatory reactions, and promoting the function of endothelial cells [179, 189, 190]. Recently, some studies also indicated that SCFAs not only induced the emergence of neurotransmitters through the systemic circulation or the vagus nerve pathway but also regulated neurotrophic factor levels, reduced neuroinflammation, and mitigated glial cell dysfunction after directly crossing the blood-brain barrier, which is crucial to the treatment of anxiety and depression [191, 192]. The effects of BAs on CAD primarily depend on their polarities. The abnormal metabolism of liposoluble BAs aggravates CAD by inducing calcium overload, activating the autonomic nervous system, regulating related receptor pathways, and triggering mitochondrial dysfunction, but the water-soluble BAs can mitigate CAD [193, 194]. The effects of BAs on anxiety and depression primarily depend on their receptors. The BAs suppress the synthesis of BDNF to aggravate anxiety and depression when targeting farnesoid X receptor, but they alleviate anxiety and depression by inhibiting neuroinflammation, oxidative stress, and nitrosation stress when targeting Takeda G-protein-coupled receptor 5 [87, 195]. Although BCAAs, as one energy substance, are beneficial for the repair of ischemic myocardial cells, the excessive elevation of BCAAs caused by their abnormal catabolism aggravates CAD by inhibiting SOD activity and assisting ROS accumulation to induce oxidative stress [196, 197]. It was acknowledged that the required amount of BCAAs that cross the blood-brain barrier contributes to enhancing the synthesis of glutamate and maintaining the balance of nitrogen in the brain to nourish neurons to mitigate anxiety and depression, but the massive BCAAs may lead to serious damage to the central nervous system to deteriorate anxiety and depression [198]. Thus, it can be seen that the metabolites of GM play an essential role in associating CAD with anxiety and depression.

So far, we identified that four kinds of commonly up-regulated bacteria (i.e., *Staphylococcus*, *Escherichia coli*, *Helicobacter pylori*, and *Shigella*) and five kinds of commonly down-regulated bacteria (i.e., *Prevotella*, *Lactobacillus*, *Faecalibacterium prausnitzii*, *Collinsella*, and *Bifidobacterium*) in CAD or anxiety and depression. The previous studies indicated that the disorder of the diversity and relative abundance of GM and the disorder of some GM-derived metabolites were expected to be the biomarkers for the clinical diagnosis of CAD or anxiety and depression [199, 200]. For instance, the reduced relative abundance of some probiotics, such as *Lactobacillus* and *Bifidobacterium*, may indicate the severe risk and poor prognosis for CAD as well as anxiety and depression [35, 201, 202]. The increase of conditioned pathogens is usually associated with a high risk of CAD or anxiety and depression [203, 204]. The elevation of TMAO promotes cholesterol accumulation, causes vascular endothelial dysfunction, and enhances the formation of atherosclerotic plaque, which has been considered one of the potential evaluation indicators for CAD [205]. The reduction of monoamine neurotransmitters, such as dopamine, norepinephrine, and serotonin, is one of the markers of anxiety and depression [206]. In addition, the decline of SCFAs, especially for propionic acid and butyric acid, affects lipid metabolism, inflammatory response, endothelial function, and synthesis and release of monoamine neurotransmitters, which may be one of the potential biomarkers of CAD or anxiety and depression [192, 207]. Moreover, the supplement of probiotics and prebiotics is expected to be one adjuvant method for the clinical treatment of CAD as well as anxiety and depression by regulating the composition and function of GM. On one hand, the adjustment to the diet helps to improve the composition and function of GM to improve the immune system, the glycolipid metabolism, and the synthesis of neurotransmitters, alleviating CAD as well as anxiety and depression. On the other hand, the supplement of probiotics is also beneficial to CAD as well as anxiety and depression. In previous studies, it has been demonstrated the supplementation of probiotics is conducive to mitigating CAD as well as anxiety and depression, such as *Lactobacillus plantarum* and *Bifidobacterium longum* [208-211]. Therefore, intensive studies on GM and its metabolites contribute to the accurate diagnosis and treatment and the improvement of the prognosis of CAD with anxiety and depression.

## Conclusion

Taken together, in CAD or anxiety and depression, four kinds of commonly up-regulated bacteria (i.e., *Staphylococcus*, *Escherichia coli*, *Helicobacter pylori*, and *Shigella*) frequently lead to the outburst of the inflammatory response, and five kinds of commonly down-regulated bacteria (i.e., *Prevotella*, *Lactobacillus*, *Faecalibacterium prausnitzii*, *Collinsella*, and *Bifidobacterium*) are usually related to the metabolic abnormality of SCFAs, BAs, and BCAAs. GM and its metabolites act as the emergent bridge between CAD and anxiety and depression. The findings of this review suggested that the effective regulation of the composition and function of GM may be novel insights and approaches for the clinical treatment of patients with both CAD and anxiety and depression.
